# 411. Comparison of clinical outcomes between early and delayed transplantation after SARS-CoV-2 infection

**DOI:** 10.1093/ofid/ofad500.481

**Published:** 2023-11-27

**Authors:** A Reum Kim, Eui Jin Chang, SeongMan Bae, Jiwon Jung, Min Jae Kim, Yong Pil Chong, Sang-Oh Lee, Sang-Ho Choi, Yang Soo Kim, Sung-Han Kim

**Affiliations:** Pusan National University Yangsan Hospital, Seoul, Seoul-t'ukpyolsi, Republic of Korea; Department of Internal Medicine, Asan Medical Center, Seoul, Korea, Seoul, Seoul-t'ukpyolsi, Republic of Korea; Asan medical center, Seoul, Seoul-t'ukpyolsi, Republic of Korea; Asan Medical Center, Seoul, Seoul-t'ukpyolsi, Republic of Korea; Asan Medical Center, Seoul, Seoul-t'ukpyolsi, Republic of Korea; Asan Medical Center, Seoul, Seoul-t'ukpyolsi, Republic of Korea; Asan Medical Center, Seoul, Seoul-t'ukpyolsi, Republic of Korea; Asan Medical Center, Seoul, Seoul-t'ukpyolsi, Republic of Korea; Asan Medical Center, Seoul, Seoul-t'ukpyolsi, Republic of Korea; Asan medical center, Seoul, Seoul-t'ukpyolsi, Republic of Korea

## Abstract

**Background:**

Thera is limited data comparing the clinical outcomes between early and delayed transplantation after SARS-CoV-2 infection. We thus compared the clinical outcomes in solid organ transplant (SOT) and hematopoietic stem cell transplant (HCT) recipients who recently experienced SARS-CoV-2 infection depending on the timing after SARS-CoV-2 infection.

**Methods:**

We retrospectively reviewed the medical records of adult patients who underwent SOT or HCT with a history of COVID-19 infection prior to transplantation at a tertiary hospital, Seoul, South Korea from January 2021 to August 2022. Patients transplanted within 3 months after COVID-19 infection were classified into the early transplantation group, and those transplanted after 3 months were classified into the delayed transplantation group.

**Results:**

A total of 53 patients who underwent SOT and 28 patients underwent HCT with a history of COVID-19 infection prior to transplantation were reviewed. Of the SOT patients, 48 (91%) were classified in the early group (median 50.0, range 12.0-89.0 days) and 5 (9%) in the delayed group (median 105.0, range 93.0-117.0 days). In the early group, 45 patients were uneventful, 2 patients had rejection, and 2 patients died. In the delayed group, 4 patients were uneventful, and 1 patient. Of the HCT patients, 19 (68%) were identified in the early group (median 34.0, range 2.0-90.0 days) and 9 (32%) in the delayed group (median 102.0, range 92.0-188.0 days). In the early group, 15 patients were uneventful, 2 patients relapsed, and 2 patients died. In the delayed group, 4 patients were uneventful, 3 patients had acute graft-versus-host disease, 1 patient relapsed, and 1 patient died. A total of 6 patients died among SOT and HCT recipients, 5 of whom died due to bacteremia caused by multidrug-resistant bacteria and 1 from acute rejection. None of the patients who underwent SOT and HCT developed COVID-19 PCR re-positivity or COVID-19 compatible symptoms after transplant.

**Figure:**
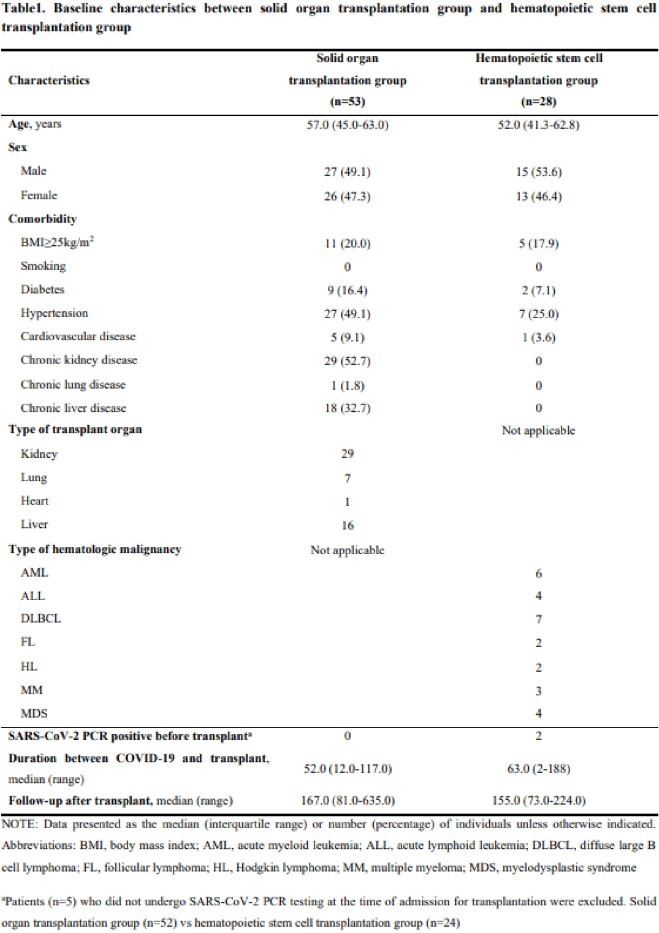

**Figure:**
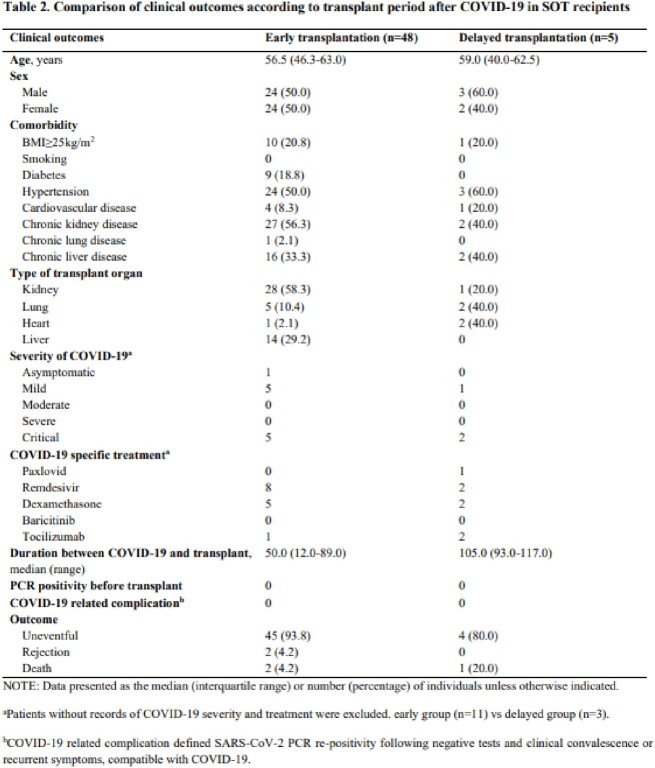

**Figure:**
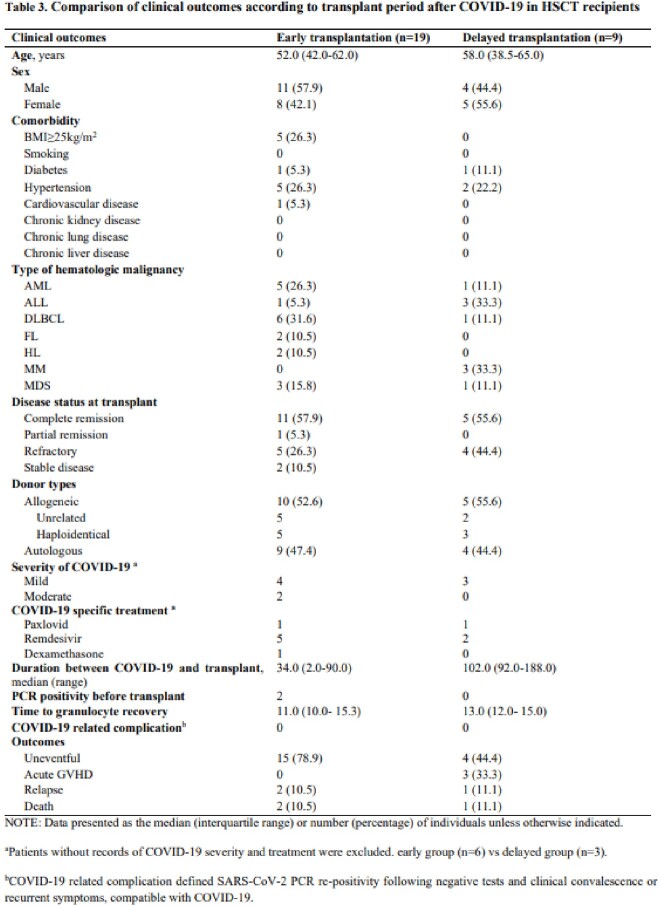

**Conclusion:**

Our data suggest that the early transplantation after SARS-CoV-2 infection may be performed without increased risk of COVID-19-associated complications.

**Disclosures:**

**All Authors**: No reported disclosures

